# Biallelic variants in *GTF3C3* result in an autosomal recessive disorder with intellectual disability

**DOI:** 10.1016/j.gim.2024.101253

**Published:** 2024-12-05

**Authors:** Lachlan De Hayr, Laura E.R. Blok, Kerith-Rae Dias, Jingyi Long, Anaïs Begemann, Robyn D. Moir, Ian M. Willis, Martina Mocera, Gabriele Siegel, Katharina Steindl, Carey-Anne Evans, Ying Zhu, Futao Zhang, Michael Field, Alan Ma, Lesley Adès, Sarah Josephi-Taylor, Rolph Pfundt, Maha S. Zaki, Hoda Tomoum, Anne Gregor, Julia Laube, André Reis, Sateesh Maddirevula, Mais O. Hashem, Markus Zweier, Fowzan S. Alkuraya, Reza Maroofian, Michael F. Buckley, Joseph G. Gleeson, Christiane Zweier, Mireia Coll-Tané, David A. Koolen, Anita Rauch, Tony Roscioli, Annette Schenck, Robert J. Harvey

**Affiliations:** 1 School of Health, University of the Sunshine Coast, Maroochydore, QLD, Australia; 2 National PTSD Research Centre, Thompson Institute, Birtinya, QLD, Australia; 3 Department of Human Genetics, Donders Institute for Brain, Cognition and Behaviour, Radboud University Medical Center, Nijmegen, The Netherlands; 4 Neuroscience Research Australia (NeuRA), Sydney, NSW, Australia; 5 Prince of Wales Clinical School, Faculty of Medicine, University of New South Wales, Sydney, NSW, Australia; 6 Institute of Medical Genetics, University of Zürich, Schlieren-Zürich, Switzerland; 7 Department of Biochemistry, Albert Einstein College of Medicine, Bronx, NY; 8 New South Wales Health Pathology Randwick Genomics, Prince of Wales Hospital, Sydney, NSW, Australia; 9 Genetics of Learning Disability Service, John Hunter Hospital, Waratah, NSW, Australia; 10 Department of Clinical Genetics, Children's Hospital Westmead, Sydney Children's Hospitals Network, Sydney, NSW, Australia; 11 Specialty of Genomic Medicine, Sydney Medical School, University of Sydney, Sydney, NSW, Australia; 12 National Research Centre, Clinical Genetics Department, Human Genetics and Genome Research Institute, Cairo, Egypt; 13 Ain Shams University, Department of Pediatrics, Cairo, Egypt; 14 Inselspital, Bern University Hospital, University of Bern, Department of Human Genetics, Bern, Switzerland; 15 Institute of Human Genetics, Universitätsklinikum Erlangen, Friedrich-Alexander-Universität Erlangen-Nürnberg, Erlangen, Germany; 16 Department of Translational Genomics, Center for Genomic Medicine, King Faisal Specialist Hospital and Research Center, Riyadh, Saudi Arabia; 17 Prince Sultan Military Medical City, Department of Pediatrics, Riyadh, Saudi Arabia; 18 Department of Neuromuscular Disorders, Institute of Neurology, University College London, London, United Kingdom; 19 University of California, Department of Neurosciences, San Diego, CA; 20 Rady Children's Institute for Genomic Medicine, San Diego, CA; 21 ITINERARE – University of Zürich Research Priority Program, Zürich, Switzerland; 22 University of Zürich and ETH Zürich, Neuroscience Center Zürich, Zürich, Switzerland

**Keywords:** GTF3C3, Intellectual disability, Minigene analysis, RNA polymerase III, Tfc4

## Abstract

**Purpose:**

This study details a novel syndromic form of autosomal recessive intellectual disability resulting from recessive variants in *GTF3C3*, encoding a key component of the DNA-binding transcription factor IIIC, which has a conserved role in RNA polymerase III-mediated transcription.

**Methods:**

Exome sequencing, minigene analysis, molecular modeling, RNA polymerase III reporter gene assays, and *Drosophila* knockdown models were utilized to characterize *GTF3C3* variants.

**Results:**

Twelve affected individuals from 7 unrelated families were identified with homozygous or compound heterozygous missense variants in *GTF3C3* including c.503C>T p.(Ala168Val), c.1268T>C p.(Leu423Pro), c.1436A>G p.(Tyr479Cys), c.2419C>T p.(Arg807Cys), and c.2420G>A p.(Arg807His). The cohort presented with intellectual disability, variable nonfamilial facial features, motor impairments, seizures, and cerebellar/corpus callosum malformations. Consistent with disruptions in intra- and intermolecular interactions observed in molecular modeling, RNA polymerase III reporter assays confirmed that the majority of missense variants resulted in a loss of function. Minigene analysis of the recurrent c.503C>T p.(Ala168Val) variant confirmed the introduction of a cryptic donor site into exon 4, resulting in mRNA missplicing. Consistent with the clinical features of this cohort, neuronal loss of *Gtf3c3* in *Drosophila* induced seizure-like behavior, motor impairment, and learning deficits.

**Conclusion:**

These findings confirm that *GTF3C3* variants result in an autosomal recessive form of syndromic intellectual disability.

## Introduction

SysNDD, an expert-curated database of gene-disease relationships in neurodevelopmental disorders,^1^ currently lists 1120 autosomal recessive, 685 autosomal dominant, and 141 X-linked genes for intellectual disability (ID) with moderate or definitive evidence. Autosomal recessive forms of ID are therefore important both because of their frequency and risk of recurrence for families. Previous isolated reports in the literature have implicated *GTF3C3* (HGNC: 4666; NCBI Gene ID: 9330), encoding general transcription factor IIIC Subunit 3, in neurodevelopmental disorders (Table 1). For example, a homozygous missense variant, c.1436A>G p.(Tyr479Cys), was found in 2 sisters with mild ID, seizures, ataxia, and dysmorphic features.^2^ A homozygous splice-site variant in *GTF3C3* c.1390+3A>G p.(Pro407_Gln500del) was also discovered in an individual with profound microcephaly, hypotonia, dysmorphic facial features, and failure to thrive.^3^ Compound heterozygous *GTF3C3* missense variants c.503C>T p.(Ala168Val) and c.2419C>T p.(Arg807Cys) were also identified^4^ in an individual with hypotonia, dysmorphic facial features, and developmental epileptic encephalopathy.

Here, we assess clinical data from 8 unpublished individuals combined with newly ascertained information from 4 published individuals to establish the contribution of *GTF3C3* to a novel syndromic form of autosomal recessive ID with variable nonfamilial facial features, severe language delay, seizures, and cerebellar/corpus callosum malformations. We also report the first detailed functional characterization of *GTF3C3* variants, using minigene assays, molecular modeling, and yeast RNA polymerase III reporter assays. Neuronal knockdown in *Drosophila* caused phenotypes recapitulating several key clinical features of this cohort, firmly establishing a causative link between *GTF3C3* variants and a syndromic form of autosomal recessive ID.

## Materials and Methods

### Cohort recruitment and clinical sequencing

An international collaboration connected through GeneMatcher^5^ assembled a cohort of individuals with *GTF3C3* variants referred from institutions in Australia, Egypt, Germany, Saudi Arabia, Switzerland, The Netherlands, the United Kingdom, and the United States. Families were assessed, informed consent was obtained, and genomic testing was performed as part of routine clinical care or as part of research studies. Clinical exome sequencing and subsequent bioinformatics analysis were performed using established procedures (see Supplemental Methods). Variants were classified utilizing the American College of Medical Genetics and Genomics and the Association for Molecular Pathology guidelines.^6^ The human sequence variants in this manuscript are described using the reference sequences NM_012086.5 (transcript) and NP_036218.1 (protein). The reference genome build GRCh37/hg19 was used for initial alignments (see Supplemental Methods), and Table 1 contains coordinates based on the latest genome build (GRCh38/hg38).

### Molecular modeling

The impact of amino acid substitutions in yeast τ131/Tfc4 (NCBI Gene ID: 852938) equivalent to human *GTF3C3* missense variants was examined in the resolved structure of the yeast TFIIIC subcomplex τA (RCSB PDB: 6YJ6)^7^ using the ChimeraX molecular graphics system.^8^ Missense variants were modeled using the Swapaa command and the Dunbrack backbone-dependent rotamer library.^9^

### Yeast RNA polymerase III reporter gene assays

The impact of amino acid substitutions in τ131/Tfc4 was examined using yeast RNA polymerase III reporter gene assays as previously described.^10,11^ See Supplemental Methods and Supplemental Tables 1 and 2 for polymerase chain reaction (PCR) primers and yeast strains used for RNA polymerase III reporter gene assays.

### Minigene assays and patient fibroblast reverse transcription-polymerase chain reaction analysis

*In vitro* splicing assays for *GTF3C3* c.503C>T p.(Ala168Val) and the exon 10 donor site variant c.1390+3A>G p.(Pro407_Gln500del) were performed using minigene analysis in transfected HEK293T cells using reverse transcription-polymerase chain reaction (RT-PCR) with vector-specific primers. The effects of this variant on mRNA splicing was also studied in cultured skin fibroblasts from patient P5 using RT-PCR. See Supplemental Methods and Supplemental Table 3 for PCR primers used for creation of *GTF3C3* minigene constructs and RT-PCR.

#### *Drosophila* stocks and maintenance

*Drosophila* stocks were maintained at room temperature on a standard *Drosophila* diet (sugar, cornmeal, and yeast) and raised at 25 °C and 70% humidity in a 12h:12h light/dark cycle unless otherwise indicated. Tissue-specific knockdown of *CG8950* (Gene ID: LOC108597775, referred to as *Gtf3c3*; Protein ID: LD44919p, referred to as Gtf3c3), the *Drosophila* ortholog of *GTF3C3*, was achieved using the UAS-Gal4 system.^12^ Three inducible UAS-RNA interference (UAS-RNAi) lines for *Gtf3c3* were obtained from the Bloomington *Drosophila* Stock Center (UAS-RNAi-1, UAS-RNAi-2) and the Vienna *Drosophila* Resource Center (UAS-RNAi-3). Details of all *Drosophila* stock lines are given in Supplemental Methods and Supplemental Table 4.

#### *Drosophila* quantitative real-time-PCR

Each line and a genetic background control were crossed to the ubiquitous Act-Gal4/TM3B Sb Tb driver to determine the relative strength of the 3 *Gtf3c3* RNAi lines and to confer knockdown. Crosses were raised at 25 °C and F1 wandering L3 larvae of the appropriate genotype were subjected to quantitative real-time PCR (qPCR) analysis (see Supplemental Methods, Supplemental Table 5 for PCR primer pairs, and Supplemental file “*Gtf3c3* qPCR raw data).

#### *Drosophila* seizure-like behavior assay

The mechanical induction seizure-like behavior assay was adapted from the protocol used by Fischer et al.^13^ Flies were reared at 25 °C or 27 °C as indicated, the latter to increase the degree of knockdown with the temperature-dependent UAS-Gal4 system.^14^ Comparisons of seizure-like behavior between genotypes and genetic background controls (see Supplemental Methods) were made using an unpaired 2-tailed *t* test with GraphPad Prism version 9.0.0 for Windows (GraphPad Software).

#### *Drosophila* habituation and fatigue assays

The light-off jump reflex and fatigue assays were conducted as described previously^15^ (see Supplemental Methods). For the light-off jump reflex, 32 flies (16 males per genotype) were simultaneously exposed to 100 light-off pulses of 15 milliseconds with a 1-second interval. The noise amplitude produced by wing vibration was measured for 500 milliseconds after each light-off pulse, and sound signals above threshold were annotated as jumps. A fly was considered to have habituated when it failed to jump for 5 consecutive light-off pulses (no-jump criterion). The last jump denotes the number of trials needed to reach the no-jump criterion (trials to criterion, TTC). Habituation per genotype was scored as the mean trials to criterion (mTTC) of all flies. For the fatigue assay, flies received 50 light-off pulses of 15 milliseconds with an increased interval of 5 seconds between the pulses, which prevents the short-lived light-off jump habituation from forming. When a fly failed to jump for 5 consecutive light-off pulses (no-jump criterion), it was considered to no longer startle due to increased fatigue, and its last jump denoted the number of trials to no-jump criterion (TTC-fatigue). The TTCs of the simultaneously measured flies of the same genotype were averaged (mTTC-fatigue).

## Results

### Missense variants in *GTF3C3* result in a neurocognitive phenotype

Clinical data for 12 probands (6 females, 6 males) are summarized in Table 1. A core neurocognitive phenotype with ID was present in all individuals. Microcephaly was present in 5 of 12 (42%). Nonfamilial facial features were present in all including hairline anomalies, broad thick eyebrows, broad nasal tip and full cheeks (Supplemental Figure 1). Seizures with varying semiology occurred in 11 of 12 (with the majority treated with anticonvulsant medication). Hypotonia, dystonia, and other abnormalities of peripheral tone or movement were common (11/12, 92%). Four of 9 (44.5%) individuals with results available from cerebral imaging had corpus callosum abnormalities, 3 of 9 (33.3%) had cortical abnormalities and 6/9 (66.6%) had cerebellar abnormalities, including cerebellar atrophy.

### *GTF3C3* variants cluster in the tetratricopeptide repeat (TPR) domains

**Figure 1:**
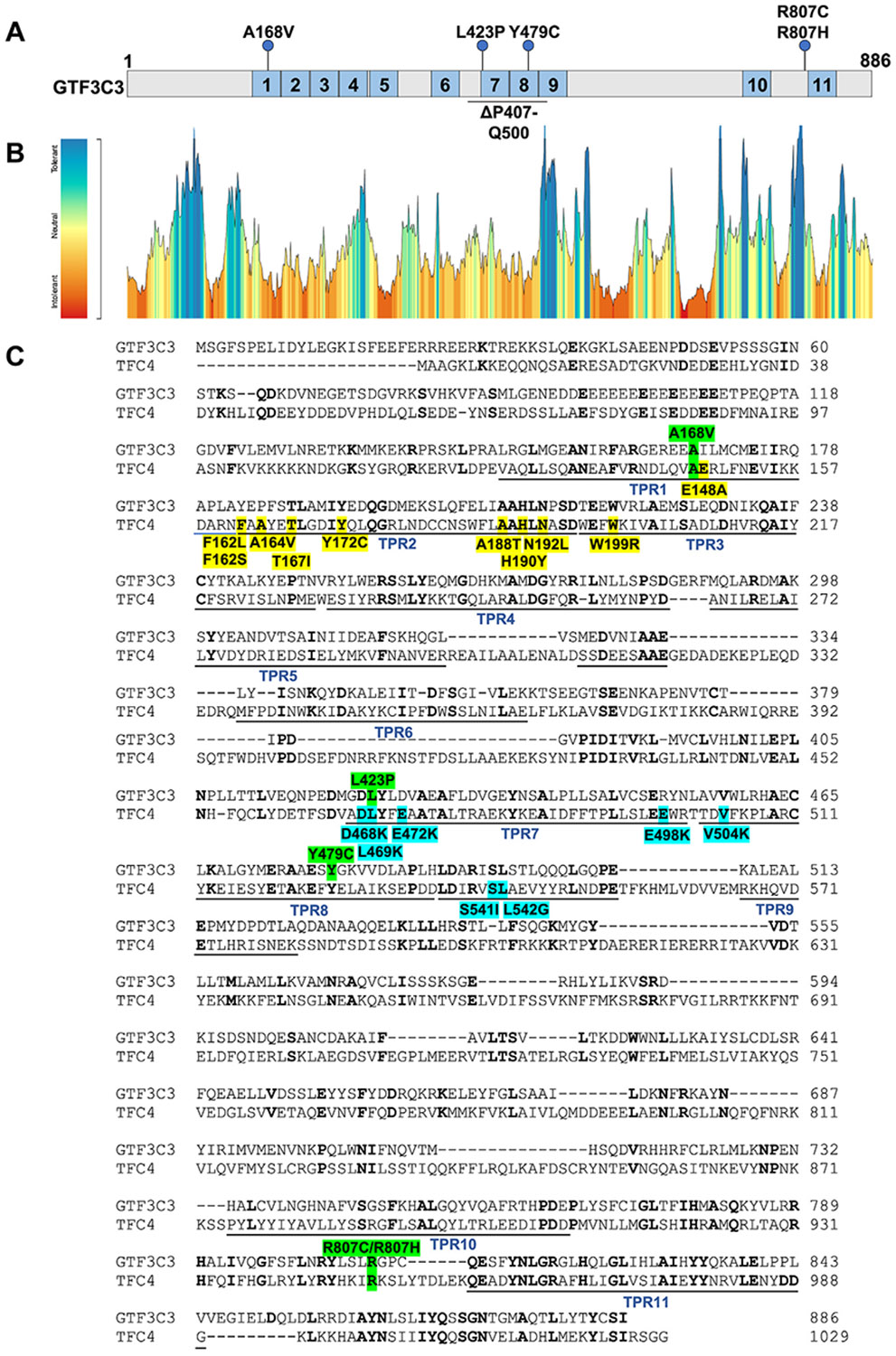
*GTF3C3* missense variants cluster in or near highly conserved tetratricopeptide repeat (TPR) domains. A. Schematic representation of the linear GTF3C3 protein (NP_036218.1; 886 amino acids) and locations of all variants in the cohort. Missense variants represented by blue circles were identified in 10 individuals, whereas the p.(Pro407_Gln500del) deletion found in 2 individuals is depicted by a solid line. The GTF3C3 protein harbors 11 tetratricopeptide repeat (TPR) domains, and missense variants are found in TPR1, TPR7, TPR8, and N-terminal of TPR11. B. A mutation tolerance landscape along the GTF3C3 protein generated by MetaDome. Genetic tolerance is computed using a missense-over-synonymous ratio over a sliding window of 11 residues based on the variation in gnomAD. Missense variants located in TPR1, TPR7, and TPR8 correlate with regions of high intolerance to missense changes. C. Alignment of human GTF3C3 protein (NP_036218.1) with the yeast ortholog τ131/Tfc4 (NP_011561.3). Identical residues are indicated in bold, human GTF3C3 protein variants in green shading, and the positions of the 11 TPR domains are underlined. Note that TPR domains 1-3 and 8-9 are contiguous. In yeast Tfc4 (τ131), mutations have been identified that affect RNA polymerase III-mediated transcription. These either increase RNA polymerase III-mediated transcription (TPRs 1-3: E148A, F162L, F162S, A164V, T167I, Y172C, H190Y, N192L, W199R, yellow shading) or decrease RNA polymerase III-mediated transcription (TPRs 6-9: L469K, E472K, V504K, S541I, L542G, cyan shading). It is noteworthy that in the human GTF3C3 protein, c.503C>T p.(Ala168Val) is located in TPR1, near gain-of-function mutations, whereas c.1268T>C p.(Leu423Pro) and c.1436A>G p.(Tyr479Cys) variants are found in TPRs 7 and 8 near loss-of-function mutations. In particular, human GTF3C3 c.1268T>C p.(Leu423Pro) is at an identical position to the yeast Tfc4 L469K loss-of-function mutation.

Eight individuals from consanguineous families had homozygous missense variants, and 2 had homozygous variants affecting a splice site. Two unrelated individuals from nonconsanguineous families had 2 different changes of the same residue, c.2419C>T p.(Arg807Cys) and c.2420G>A p.(Arg807His) (Table 1). Variant c.503C>T p.(Ala168Val) was recurrent in 7 individuals (2 unrelated individuals and 5 siblings in 2 families). All *GTF3C3* missense variants were scored as damaging by combined annotation dependent depletion score scores between 24 to 35, ClinPred scores above 0.98, REVEL scores higher than 0.534, and AlphaMissense scores between 0.5628 to 0.9969, consistent with pathogenicity (Supplemental Table 6). In silico splicing scores for *GTF3C3* variants using Alternative Splice Site Predictor and Splice AI were consistent with c.503C>T p.(Ala168Val) resulting in the gain of a cryptic splice donor site (Supplemental Table 7). The same bioinformatic tools predicted the loss of a donor site for the known *GTF3C3* splice variant c.1390+3A>G p.(Pro407_Gln500del). The majority of missense variants were located in the conserved TPR motifs (Figure 1A-C, Supplemental Figure 2), regions that are intolerant to missense variation in MetaDome^16^ (Figure 1B). The only exceptions were c.2419C>T p.(Arg807Cys) and c.2420G>A p.(Arg807His), which are located upstream of TPR11 and were classified as neutral (Figure 1A-C). All variants were unreported in ClinVar. Three of the 4 compound heterozygous variants: c.1268T>C p.(Leu423Pro), c.2419C>T p.(Arg807Cys), and c.2420G>A p.(Arg807His) were present in gnomAD in the heterozygous state, but at low frequencies, consistent with a rare autosomal recessive condition.

### Molecular modeling

**Figure 2:**
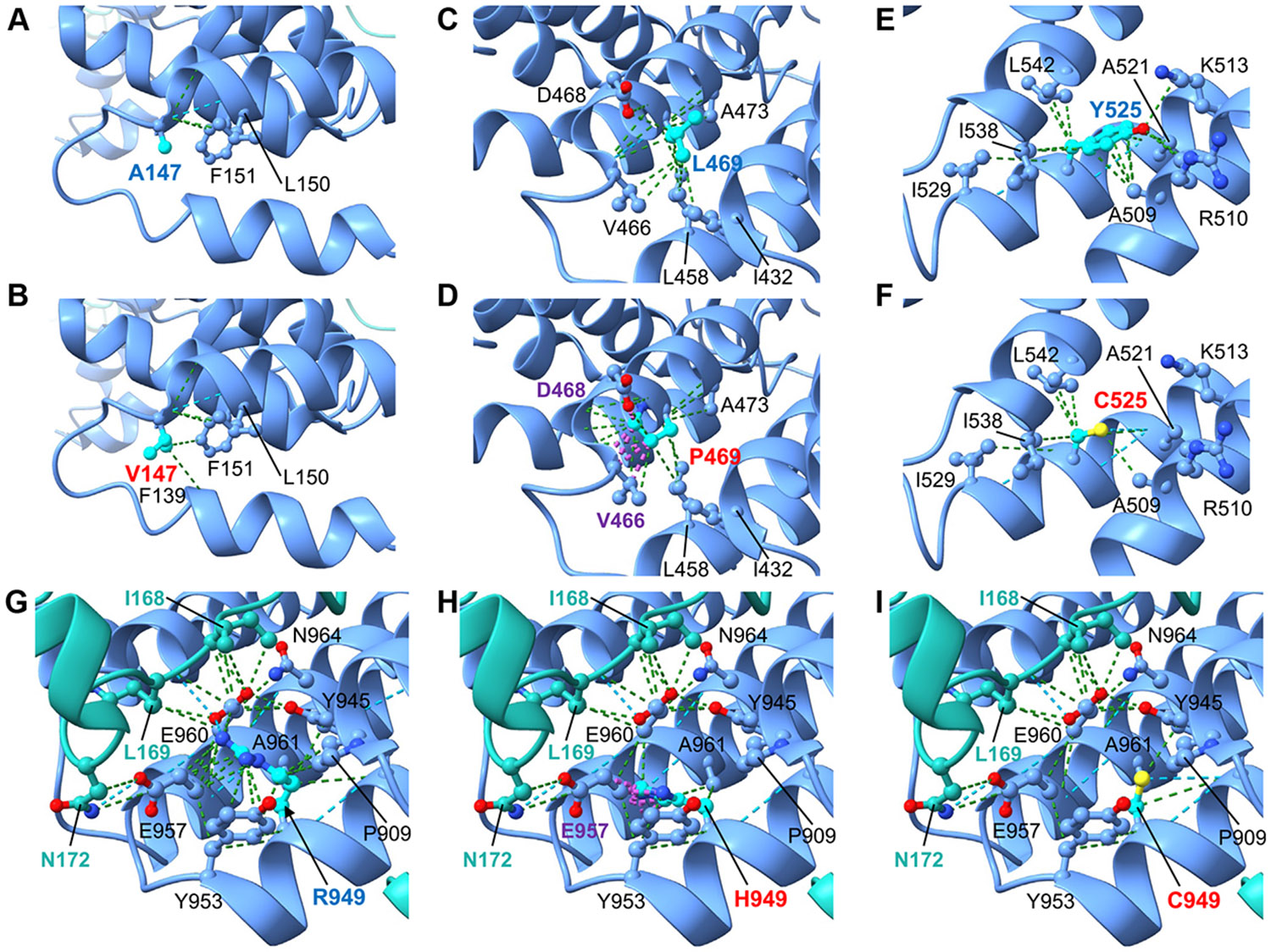
Molecular modeling of *GTF3C3* variants in the structure of yeast τ131/Tfc4. A and B. The substitution A147V in yeast τ131/Tfc4 TPR1 (human c.503C>T p.(Ala168Val) equivalent) creates some additional minor contacts with neighboring residues F139 and F151. C and D. L469 in yeast τ131/Tfc4 has a key role in the stabilization of TPR7, making intra-helix contacts with V466 and A473 and contacts with L458 and I432 on an adjacent α-helix. The L469P substitution (human c.1268T>C p.(Leu423Pro) equivalent) generates several clashes with residues V466 and D468 and is predicted to destabilize intrahelix contacts in TPR7. E and F. Y525 in yeast τ131/Tfc4 TPR8 plays a key role in stabilizing the organization of TPR domains through numerous contacts, including the highly conserved residues A509, R510, K513, and A521 within TPR8, as well as I538, I539, and L542 in TPR9. The Y525C substitution (human c.1436A>G p.(Tyr479Cys) equivalent) results in the loss of the aromatic ring and the majority of contacts with A509, R510, K513, and A521 within TPR8. G-I. R949 in yeast τ131/Tfc4 is located just before TPR11 and forms numerous contacts with neighboring residues including P909, Y945, Y953, E957, and E960, A961 and N964 in TPR11. The R949H substitution (human c.2420G>A p.(ArgR807His) equivalent) results in the loss of the hydrogen bonds and contacts with E960 and E957, as well as introducing clashes with E957. E957 forms part of an interface formed by E957 and E960 with residues I168, L169, and N172 in yeast τ95/Tfc1 (turquoise). By contrast, the R949C substitution (human c.2419C>T p.(ArgR807Cys) equivalent) results in the loss of preexisting hydrogen bonds and contacts within TPR11 (eg, with Y953, E957, and E960).

τ131/Tfc4 is the yeast ortholog of the GTF3C3 protein and has a similar domain architecture,^17^ including 11 TPR motifs (Figure 1C). The amino acids corresponding to Ala168, Leu423, Tyr479, and Arg807 are also completely conserved in yeast τ131/Tfc4 (Figure 1C), corresponding to Ala147, Leu469, Tyr525, and Arg949, respectively. The impact of the corresponding amino acid substitutions in τ131/Tfc4 was examined in the resolved structure of the yeast TFIIIC subcomplex τA (RCSB PDB: 6YJ6).^7^ Alanine 168 in TPR1 makes backbone contacts with F151 and L150 (Figure 2A). The c.503C>T p.(Ala168Val) substitution (A147V in yeast Tfc4) did not result in any predicted conformational changes, although some additional contacts were created with F151 and F139 in a parallel *α*-helix (Figure 2B). By contrast, leucine 423 makes intra helix-stabilizing contacts with V466 and A473 in TPR7 and I432 and L458 located on a parallel helix (Figure 2C). The substitution c.1268T>C p.(Leu423Pro) (L469P in yeast τ131/Tfc4) generates clashes with neighboring residues V466 and D468 (Figure 2D). Because proline substitutions are also known to introduce kinks in helices^18^ this substitution is predicted to destabilize intrahelix contacts in TPR7.

The substitution c.1436A>G p.(Tyr479Cys) (Y525C in yeast τ131/Tfc4) affects a tyrosine residue that stabilizes parallel *α*-helices via contacts with residues A509, R510, K513, A521, and I529 in TPR8 and I538 and L542 in TPR9 (Figure 2E). For Y525C, the cysteine maintains original contacts with I529, I538, and L542 but loses other contacts with A509, R510, K513, and A521 (Figure 2F). This loss of numerous contacts with TPR8 is likely to result in significant destabilization of the GTF3C3 protein structure. Lastly, for c.2419C>T p.(Arg807Cys) and c.2420G>A p.(Arg807His), the positively charged residue R949 in τ131/Tfc4 forms key contacts within TPR11 (eg, P909, Y945, Y953, E957, E960, A961, and N964). Two of the most prominent interactions include contacts and hydrogen bonds with E957 and E960, which, in turn, interact directly with I168, L169, and N172 in yeast τ95/Tfc1 (Figure 2G). The substitution c.2420G>A p.(Arg807His) (R949H in τ131/Tfc4) results in the loss of the hydrogen bonds and contacts with E960 and E957, as well as introducing clashes with E957, which forms part of the interface with N172 in yeast τ95/Tfc1 (Figure 2H). In contrast, the c.2419C>T p.(Arg807Cys) equivalent (R949C) results in the loss of the majority of preexisting hydrogen bonds and contacts with TPR11 (Figure 2I). Both c.2420G>A p.(Arg807His) and c.2419C>T p.(Arg807Cys) are therefore predicted to destabilize protein-protein interactions of GTF3C3 with GTF3C5, the human equivalent of yeast τ95/Tfc1.

### Yeast RNA polymerase III reporter gene assays reveal that the majority of *GTF3C3* missense variants result in a loss of function

**Figure 3:**
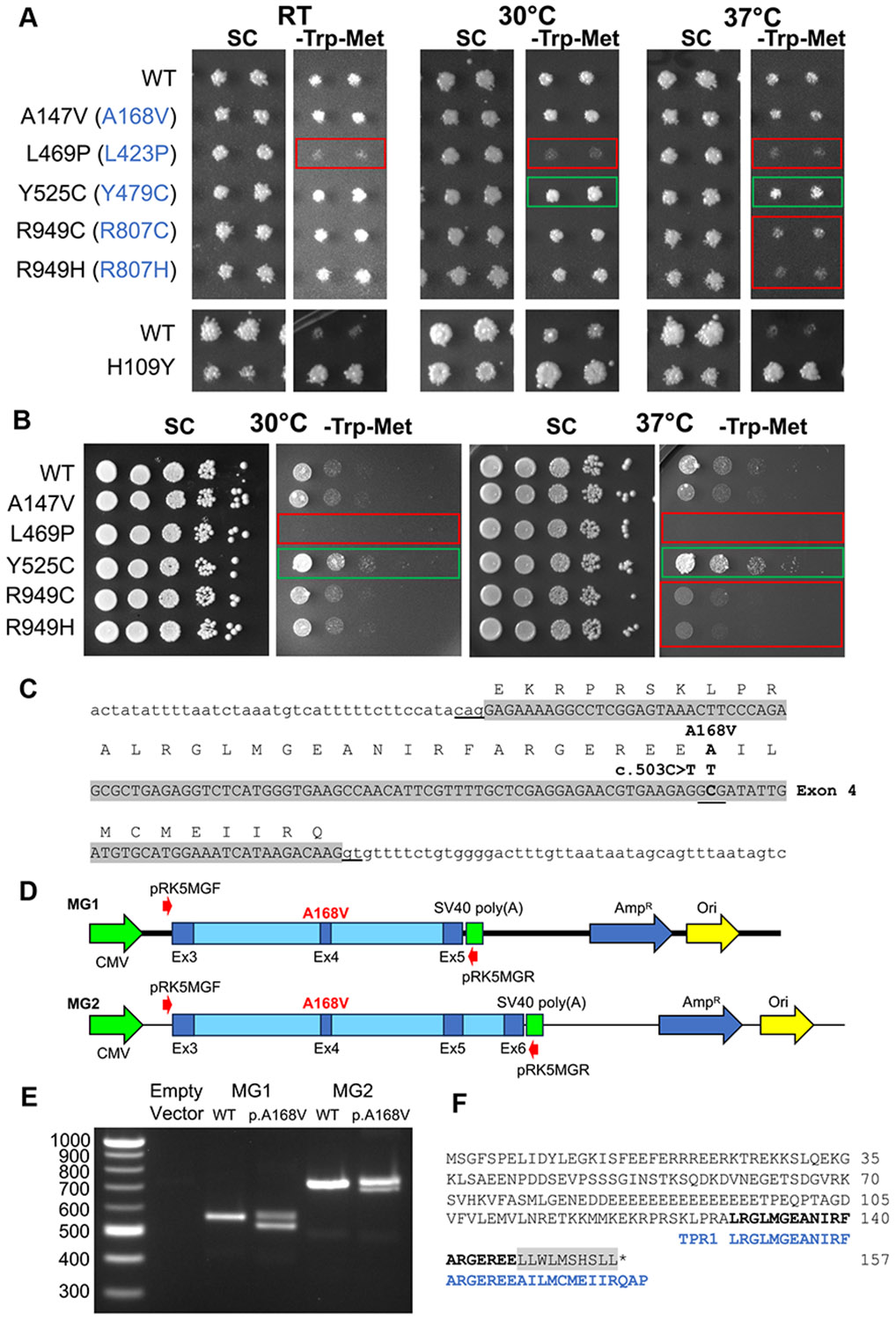
*GTF3C3* missense variants cause altered growth patterns in yeast RNA polymerase III reporter gene assays, or missplicing in minigene assays. A and B. Yeast RNA polymerase III reporter assay. Yeast strains that contain either a wild-type or mutated Tfc4 (Tfc4^A147V^, Tfc4^L469P^, Tfc4^Y525C^, Tfc4^R949C^, and Tfc4^R949H^) were assayed for growth phenotypes. (A) Replica plate assay of mutant and wild-type strains printed onto synthetic complete (SC) or suppression media (SC-Trp-Met) at room temperature (RT), 30 °C or 37 °C for 3 days. Transcription of the polymerase III suppressor tRNA, *sup9eA19-supS1*, leads to readthrough of the amber stop codon in the yeast *trp1-1* and *met8-1* auxotrophic markers to allow growth on suppression media without tryptophan and methionine (SC-Trp-Met). Note that Tfc4^L469P^ leads to reduced growth on suppression media (SC-Trp-Met) at all temperatures (red box), whereas Tfc4^Y525C^ leads to increased growth (green box). Tfc4^R949C^ and Tfc4^R949H^ appear to have a temperature-dependent phenotype, with reduced growth on suppression media seen at 37 °C. Lower panels: Replica images of the yeast Tfc4 gain-of-function mutant Tfc4^H190Y^, grown contemporaneously with the humanized yeast strains provides a comparison with the gain-of-function/suppression phenotype of Tfc4^Y525C^. Panel (B) represents a 10-fold serial dilution series of the experiments shown in Panel (A), which more clearly delineates the mild gain of function for Tfc4^Y525C^, loss of function for Tfc4^L469P^, and temperature-sensitive loss of function for Tfc4^R949C^ and Tfc4^R949H^. C-F. Minigene analysis. (C) Sequence of *GTF3C3* Exon 4, showing the location of the missense variant c.503C>T p.(Ala168Val). (D) Constructs pRK5 (empty vector), pRK5 *GTF3C3* MG1 (exons 3-5) and pRK5 *GTF3C3* MG2 (exons 3-6) were transfected into HEK293 cells. (E) PCR amplifications from first-strand cDNA with primers pRK5MGF and pRK5MGR (Supplemental Table 3). Expected amplification products were observed for empty vector (no product) and wild-type MG1 and MG2, but the c.503C>T p.(Ala168Val) variant resulted in missplicing in both minigene constructs. (F) Sanger DNA sequencing confirmed the use of the exon 4 cryptic splice donor site, resulting in loss of part of exon 4 and a frameshift (gray highlighting) and truncation of the GTF3C3 protein before the end of TPR1.

A yeast nonsense suppressor assay was utilized to assess the effect of human *GTF3C3* mutants on RNA polymerase III-mediated transcription. In yeast, the ortholog of the GTF3C3 protein, τ131/Tfc4, is essential for yeast viability (Supplemental Figure 3).^17^ The yeast strain supRM, which utilizes the nourseothricin N-acetyl transferase selection marker to completely delete Tfc4 was used to assess the activity of wild-type and mutant Tfc4. The supRM strain also has an engineered integrated suppressor tRNA, sup9eA19-supS1, to allow readthrough of amber stop codons. Any increase in transcription by RNA polymerase III leads to more sup9eA19-supS1 tRNA expression, more readthrough, and consequently, more growth on -Trp/-Met media (Figure 3A). Tfc4^A147V^, equivalent to c.503C>T p.(Ala168Val), showed no change in suppressor activity at any temperature, consistent with this variant having no effect on RNA polymerase III-mediated transcription in yeast. In contrast, Tfc4^L469P^, equivalent to c.1268T>C p.(Leu423Pro), resulted in reduced growth on suppression media (SC-Trp-Met) at all temperatures, consistent with a loss of function. Interestingly, Tfc4^Y525C^, equivalent to c.1436A>G p.(Tyr479Cys), resulted in increased growth, consistent with a mild gain of function (Figure 3A), an effect that was most evident in serial dilutions of the yeast (Figure 3B). Lastly, Tfc4^R949C^ and Tfc4^R949H^, equivalent to c.2419C>T p.(Arg807Cys) and c.2420G>A p.(Arg807His), showed a temperature-dependent loss-of-function phenotype, with reduced growth on suppression media seen at 37 °C compared with 30 °C (Figure 3A and B).

### The recurrent *GTF3C3* variant c.503C>T p.(Ala168Val) causes missplicing

Given the lack of apparent impact of the recurrent variant c.503C>T p.(Ala168Val) in molecular modeling (Figure 2) and yeast RNA polymerase III reporter gene assays (Figure 3A and B), and results from splicing prediction tools Alternative Splice Site Predictor and SpliceAI (Supplemental Table 7), the impact of this variant on *GTF3C3* mRNA splicing were assessed in minigene assays (Figure 3C-F). The c.503C>T change introduces a cryptic donor site (AGgt) within exon 4 that is identical to the canonical donor site at the end of wild-type exon 4 (Figure 3C). Constructs pRK5 (empty vector), pRK5 *GTF3C3* MG1 (exons 3-5), and pRK5 *GTF3C3* MG2 (exons 3-6) (Figure 3D) were transfected into HEK293 cells, and PCR amplifications were performed from first-strand cDNA with vector-specific primers pRK5MGF and pRK5MGR (Figure 3D; Supplemental Table 1). No products were observed for the empty vector. However, expected product sizes of 562 bp and 728 bp were produced for wild-type minigene constructs MG1 and MG2. Equivalent constructs containing the c.503C>T change resulted in RNA missplicing for both minigene constructs (Figure 3E). Consistent with the cryptic donor site having a consensus sequence identical to the normal exon 4 donor site, missplicing was only seen in a proportion of *GTF3C3* transcripts (Figure 3E). Sanger DNA sequencing confirmed the use of the cryptic splice donor site resulted in the loss of part of exon 4 and a frameshift causing truncation of the GTF3C3 protein before the end of TPR1 (Figure 3F). To further investigate the impact of the c.503C>T change on splicing, experiments were performed with mRNA isolated from cultured skin fibroblasts from patient P5 and a control individual (Supplemental Figure 4). Following treatment with the translation inhibitor cycloheximide to prevent nonsense-mediated mRNA decay, a similar incomplete aberrant splicing pattern was observed by RT-PCR in the patient cells (Supplemental Figure 4A), and a 34-bp deletion was confirmed by Sanger sequencing of the PCR products (Supplemental Figure 4B).

### A combined effect of patient P5 compound heterozygous variants on GTF3C3 protein levels

Analysis of GTF3C3 protein levels by western blotting in patient P5 fibroblast samples compared with 3 independent controls unveiled a significant reduction in overall protein levels (Supplemental Figure 4C), supporting the conclusion that aberrant splicing and consequent nonsense-mediated decay of c.503C>T transcripts translates to a reduction in GTF3C3 protein levels. Although GTF3C3 protein levels normalized to β-actin and GAPDH showed some variability between control cell lines, GTF3C3 protein levels in patient 5 were consistently reduced by >50% compared with each control in all 3 experiments. Because our minigene and RT-PCR data indicate that the effect of c.503C>T on mRNA splicing is leaky (Figure 2, Supplemental Figure 4), this suggests that the c.2419C>T p.(Arg807Cys) variant also impairs GTF3C3 protein stability, perhaps because of impaired GTF3C3-GTF3C5 interactions as predicted by our structural modeling (Figure 2).

### *GTF3C3* exon 10 donor site variant c.1390+3A>G also causes missplicing

Lastly, minigene assays were performed as a control for the *GTF3C3* exon 10 donor site variant c.1390+3A>G in a construct (pRK5 *GTF3C3*-MG4) containing exons 9-12 (Supplemental Figure 5). Consistent with multiple PCR product sizes observed in patient cDNA,^3^ several new splice products were observed for the pRK5 *GTF3C3*-MG4 c.1390+3A>G minigene that were not observed in the equivalent wild-type construct (Supplemental Figure 5). These included the use of a cryptic exonic donor site that results in an in-frame deletion of 87 bp, encompassing parts of TPR7 and TPR8 (splice isoform 1), and an intronic cryptic donor site resulting in protein truncation (splice isoform 2). Hence, at least 2 *GTF3C3* variants result in complex splicing defects.

#### *Drosophila Gtf3c3* knockdown models

**Figure 4:**
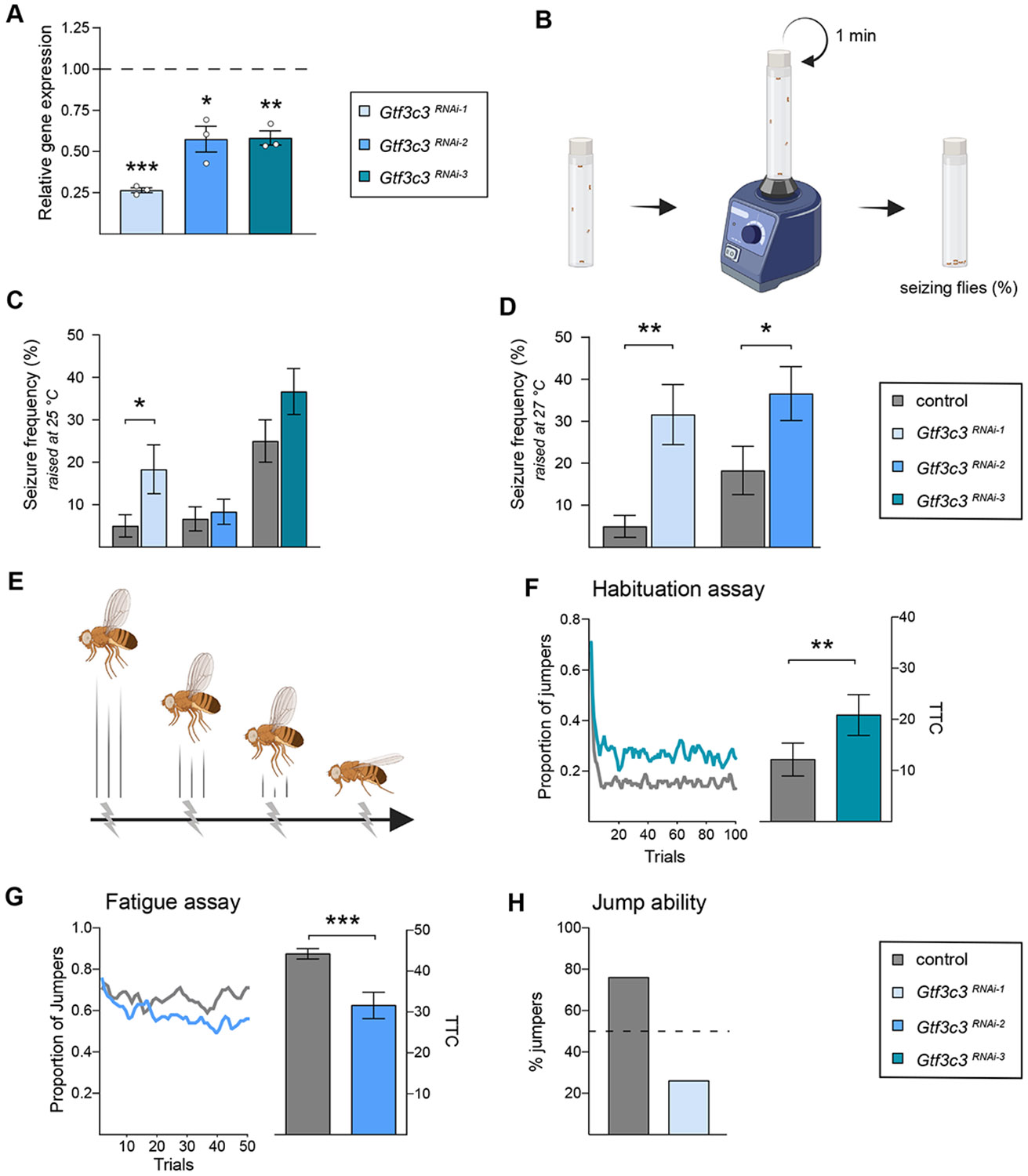
*Gtf3c3* knockdown leads to increased seizure-like behavior, deficits in habituation learning and motor impairment in *Drosophila*. A. Relative Gtf3c3 mRNA levels in third instar larva upon ubiquitous *Gtf3c3* knockdown (*Actin-Gal4>UAS-RNAi*) with *UAS-RNAi-1* to *−3*, normalized to their respective genetic background controls (*Actin-Gal4/+*, dashed line). Statistical significance of 2^-ΔΔCT values was calculated using an unpaired 2-tailed *t* test. Induction of each of the 3 lines induces a significant reduction in *Gtf3c3* mRNA levels. B. Schematic representation of the paradigm used to assess seizure-like behavior upon mechanical induction. C and D. Frequency of seizure-like behavior observed within 5 seconds after mechanical induction shown by *Gtf3c3^RNAi^* (*nSyb-Gal4>UAS-RNAi*) animals and their respective genetic background controls (*nSyb-Gal4/+*). All experiments were conducted with 5 animals per vial, *n* = 12 vials per genotype. Statistical significance was calculated using an unpaired two-tailed *t* test. (C) Raised at a standard temperature of 25 °C, seizure-like behavior was significantly increased in *Gtf3c3^RNAi-1^* animals compared with controls. Note the variable seizure frequency shown by the different genetic backgrounds. (D) Raised at a more stringent temperature of 27 °C, seizure-like behavior was significantly increased in *Gtf3c3^RNAi-1^* and *Gtf3c3^RNAi-2^ flies*. E. Simplified scheme of habituation learning in the light-off jump habituation paradigm. In reality, not the amplitude but the frequency of jumps in the tested population wanes. F and G. Results of subjecting *Gtf3c3^RNAi^* animals and their respective controls to the light-off jump paradigms. (F) Pan-neuronal *Gtf3c3* knockdown using UAS-RNAi-3 (*n* = 135, in turquoise) leads to deficits in habituation learning, evidenced by an increase in mTTC (*P* = 8.77E^−4^) compared with the genetic background control (*n* = 111, in gray). (G) Pan-neuronal knockdown of *Gtf3c3* using the *UAS-RNAi-2* construct (*n* = 85, in blue) significantly reduced their jump fitness, as revealed during the fatigue assay by the inability to maintain a constant jump response and the significantly lower mean trials to the no-jump criterion (mTTC-fatigue) (*P* = 1.85E^−5^) compared with genetic background controls (*n* = 87, in gray). H. Pan-neuronal knockdown of *Gtf3c3* with the strong *UAS-RNAi-1* construct severely impairs their initial jump response in the habituation assay (26%, *n* = 50 jumpers of 192 tested, in light blue), compared with the control (76%, *n* = 134 of 176 tested, in gray), which precludes proper assessment of habituation. Statistical significance in G and H was calculated by via a linear model regression analysis on logarithmic transformed mTTC values. Bar charts depict mean ± SEM. * *P* ≤ .05, ** *P* ≤ .001, *** *P* ≤ .0001. Panels (B) and (D) were created using BioRender.

The *Drosophila melanogaster* genome encodes a single ortholog of *GTF3C3*. This previously uncharacterized gene carries the identifier CG8950 and is hereafter referred to as *Gtf3c3*. Human and *Drosophila* GTF3C3/Gtf3c3 proteins share 30% amino acid identity and contain a similar TPR domain structure (Supplemental Figure 6). Genome-wide expression data in FlyBase indicate that *Drosophila Gtf3c3* is widely expressed, although at low levels. Single-cell sequencing data indicate that in the nervous system, *Gtf3c3* expression is scattered across neurons.^19^ *Gtf3c3* loss-of-function models were generated using the binary UAS-Gal4 system^12^ because most of the identified *GTF3C3* variants lead to loss of function. Three inducible RNA interference (RNAi) lines targeting *Gtf3c3* were recruited, which were termed *Gtf3c3 UAS-RNAi-1, UAS-RNAi-2*, and *UAS-RNAi-3*, together with their corresponding genetic background control lines (Supplemental Table 4). To assess the efficiency and relative strength of the 3 RNAi constructs, each line and their respective genetic background lines were crossed to a ubiquitously expressed promoter line (Act-Gal4). The remaining mRNA levels were determined in F1 wandering L3 larvae by quantitative RT-PCR. Each of the 3 RNAi constructs induced a significant knockdown of *Gtf3c3* mRNA. The remaining expression level of *Gtf3c3* mRNA was 26.6% in *Gtf3c3*^RNAi-1^ (*P* = 5.68E^−5^), 57.6% in *Gtf3c3*^RNAi-2^ (*P* = .015), and 58.3% in *Gtf3c3*^RNAi-3^ (*P* = .001) animals, relative to the level of expression in the respective genetic background control (Figure 4A, Supplemental Table 4, Supplemental file “Gtf3c3 qPCR raw data”).

### Loss of neuronal Gtf3c3 protein results in seizure-like behaviors in *Drosophila*

Knockdown of *Gtf3c3* was induced specifically in *Drosophila* neurons using pan-neuronal drivers to support the causal link between *GTF3C3* loss of function and the clinical phenotypes observed in our patient cohort. Related to seizures seen in 11/12 patients, we first addressed whether *Gtf3c3* loss of function confers susceptibility to seizures. In *Drosophila*, seizure-like behavior can be triggered by strong mechanical stimulation and can manifest as paralysis^20^ and uncontrolled movement of wings and legs while flies lay on their back^21,22^ (Figure 4B). When raised at standard conditions (25 °C), pan-neuronal *Gtf3c3* knockdown with the strongest RNAi construct, *Gtf3c3 UAS-RNAi-1*, led to a significant increase in seizure frequency compared with background controls, whereas *UAS-RNAi-2* or *UAS-RNAi-3* did not (Figure 4C). Given the autosomal recessive nature of the human variants and the only mild knockdown induced by the 2 constructs, it is likely that the elicited knockdown is insufficient to induce the seizure-like phenotype. Because the efficiency of the UAS-Gal4 system can increase with temperature,^12^ flies with induced *UAS-RNAi-1* and *UAS-RNAi-2* constructs (omitting *UAS-RNAi-3* because of the high baseline seizure frequency of the genetic background, Figure 4C) were reared at 27 °C instead of 25 °C. Under these more stringent conditions, knockdown with *Gtf3c3 UAS-RNAi-2* also caused a significant increase in seizure-like behavior compared with its genetic background control. *Gtf3c3* knockdown with *Gtf3c3 UAS-RNAi-1* further increased seizure frequency and significance of the phenotype (Figure 4D).

### *Drosophila Gtf3c3* knockdown causes deficits in habituation learning and motor impairment

Given the motor and cognitive impairments shared by all patients in this cohort, the role of *Gtf3c3* in motor performance and habituation was evaluated. Habituation learning is an evolutionary-conserved form of nonassociative learning that causes an initial response to a repeated irrelevant stimulus to gradually wane.^23^ Habituation deficits, reflecting an inability to adapt and reduce the resulting responses, have been reported in ID and autism spectrum disorder (ASD) cohorts and have been found to characterize more than a 100 *Drosophila* models of ID/ASD.^24,25^ *Gtf3c3* knockdown models were subjected to the light-off jump habituation assay (Figure 4E), in which motor performance and habituation are simultaneously assessed. In this assay, an escape response is initially triggered by a light-off pulse mimicking an approaching predator and subsequently habituates as no attack occurs.

*Gtf3c3*^RNAi-3^ animals initiated jumping as efficiently as their genetic background flies but habituated more slowly and incompletely to the repeated stimulus (Figure 4F). A total of 20.8 trials were required on average to habituate (*n* = 135) compared with 12.1 trials required by their controls (*n* = 111), revealing a significant deficit in habituation (*P* = 8.77E^−04^). *Gtf3c3*^RNAi-2^ animals were also able to jump (Supplemental Figure 7) but stopped jumping earlier than their genetic background controls during the habituation assay, raising the possibility that they either show improved learning or, more likely, motor fatigue (Supplemental Figure 7). *Gtf3c3*^RNAi-2^ flies were analyzed in the fatigue assay, a modified habituation assay in which the time between the light-off pulses is increased to 5 seconds, preventing short-lived light-off jump habituation, to distinguish between motor and learning impairment. Figure 4G shows that *Gtf3c3*^RNAi-2^ animals were unable to maintain their jump response throughout the course of the experiment during the fatigue assay (*Gtf3c3*^RNAi-2^ mTTC-fatigue = 31.6, compared with the control with mTTC-fatigue = 44.2, *P* = 1.38E^−05^), consistent with motor fatigue rather than improved habituation. Pan-neuronal knockdown using the strong *Gtf3c3 UAS-RNAi-1* line strongly impaired the ability of the flies to jump to the light-off stimulus. Only 26% showed a jump response to 1 of the first 5 trials (Figure 4H), precluding assessment of habituation and fatigue. The complete set of acquired habituation, fatigue, and jump ability data is shown in Supplemental Figure 7.

Given that 5 of 12 individuals in our cohort exhibited microcephaly, we also evaluated whether loss of *Gtf3c3* affects *Drosophila* brain size. We dissected, labeled and measured brains of wandering L3 larvae from the 3 *Gtf3c3* ubiquitous knockdown models. This did not reveal significant changes in brain lobe area or brain width, compared with the genetic background controls (Supplemental Figure 8).

## Discussion

This study describes 12 individuals from 7 families with a syndromic form of ID, including non-familial facial features, movement and tone impairments, seizures, and brain malformations affecting the corpus callosum and cerebellum. All individuals had rare biallelic variants in *GTF3C3*. Molecular modeling, RNA polymerase III reporter gene assays and minigene analyses were utilized to characterize *GTF3C3* variants, and *Drosophila* knockdown models provided independent support linking *GTF3C3* to the observed clinical phenotypes. Consistent with disruptions in intra- and intermolecular interactions identified by molecular modeling, RNA polymerase III reporter assays demonstrated that missense variants corresponding to human *GTF3C3* c.1268T>C p.(Leu423Pro), c.2419C>T p.(Arg807Cys), and c.2420G>A p.(Arg807His) resulted in loss of function, whereas c.1436A>G p.(Tyr479Cys) resulted in a mild gain of function. Minigene analysis and expression analyses in patient fibroblasts confirmed that the recurrent variant c.503C>T p.(Ala168Val) introduced a cryptic donor site into exon 4, resulting in mRNA missplicing and reduced GTF3C3 protein levels. Consistent with the clinical features of our cohort, neuronal loss of *Gtf3c3* in *Drosophila* induced seizure-like behavior, motor impairment, and deficits in habituation learning, a fundamental form of cognitive functioning. Taken together, this evidence firmly establishes biallelic variants in *GTF3C3* as a cause of autosomal recessive syndromic ID.

Human TFIIIC consists of a 6-subunit protein complex, which is organized into two 3-subunit subcomplexes: τA (GTF3C3, GTF3C5, and GTF3C6 proteins) and τB (GTF3C1, GTF3C2, and GTF3C4 proteins)^26^ that bind 2 DNA motifs. These A-box and B-box motifs are variably positioned relative to one another, separated by 30 to 67 base pairs. Once TFIIIC is anchored to DNA via tB binding to the B-box, the τA subcomplex, connected by a ~550-amino acid flexible linker to τB, is thought to locate the A-box motif by a fly-casting mechanism.^27^ Known protein interactors of GTF3C3 are the TFIIIC subunit GTF3C5, which binds the A-box motif,^28^ GTF3C1,^29^ the TFIIIB components TBP and Brf1,^26^ and RNA polymerase III. Interestingly, defects in these GTF3C3-interacting proteins are also known to cause neurological disorders, including syndromic forms of ID. For example, *de novo* expansion of a CAG repeat in *TBP* has been identified^30,31^ in spinocerebellar ataxia 17, characterized by ataxia, dementia, and involuntary movements, including chorea and dystonia. Variants in *BDP1* have also been linked to hereditary hearing loss.^32^ Of note, biallelic variants in *BRF1* linked to cerebellofaciodental syndrome have significant clinical overlap with this *GTF3C3* cohort—ID, abnormal facial and dental findings, and cerebellar hypoplasia.^33^ Lastly, the GTF3C3 interactor GTF3C5 has been linked recently to a multisystem developmental disorder with features, including growth retardation, developmental delay, intellectual disability, dental anomalies, cerebellar malformations, delayed bone age, skeletal anomalies, and facial dysmorphism.^34^ It is also noteworthy that in cortical neurons, silencing of *GTF3C5* mimics the effects of chronic depolarization, inducing a dramatic increase of both dendritic length and branching.^35^

Missense variants in TPRs 1-3 of yeast τ131/Tfc4 can either increase or decrease RNA polymerase III-mediated transcription^11,29,36^ (Figure 1). Dominant gain-of-function variants are thought to stabilize intramolecular interactions in τ131/Tfc4 that promote Brf1 binding and TFIIIB recruitment,^11,37,38^ a limiting step in transcription by RNA polymerase III. Despite the close proximity of c.503C>T p.(Ala168Val) to this cluster of mutations (Figure 1), molecular modeling and RNA polymerase III reporter gene assays indicated that A147V in yeast τ131/Tfc4 did not result in functional alterations in reporter gene assays (Figure 3). Bioinformatic, minigene, and patient fibroblast analysis were however consistent with c.503C>T p.(Ala168Val) introducing a cryptic donor site into exon 4, which caused leaky aberrant splicing of *GTF3C3* transcripts. These findings add weight to the emerging evidence base that apparent missense changes contribute to pathogenicity through splicing effects more often than recognized.^39,40^

Molecular modeling and RNA polymerase III reporter gene assays strongly suggest that the *GTF3C3* variants c.1268T>C p.(Leu423Pro) (TPR7), c.2419C>T p.(Arg807Cys), and c.2420G>A p.(Arg807His) are loss-of-function variants. Missense mutants of yeast τ131/Tfc4 in TPRs 6-9 typically decrease RNA polymerase III-mediated reporter gene transcription by compromising Brf1 and Bdp1 binding.^41^ Indeed, c.1268T>C p.(Leu423Pro) is localized at an identical position to the yeast τ131/Tfc4 mutation L469K, which impairs Bdp1 binding *in vitro* and reporter gene activity *in vivo*.^29,41^ Similarly, τ131/Tfc4 mutants equivalent to *GTF3C3* c.2419C>T p.(Arg807Cys) and c.2420G>A p.(Arg807His) are temperature-sensitive loss-of-function variants in yeast and are predicted to disrupt interactions with the GTF3C5 protein (equivalent to yeast τ95/TFC1) based on the cryogenic electron microscopy structure.^7^ By contrast, although molecular modeling predicted that c.1436A>G p.(Tyr479Cys) (TPR8) would destabilize the GTF3C3 protein, the RNA polymerase III reporter gene assay showed a mild gain-of-function phenotype. Our findings indicate that Tyr479 occupies a conserved functionally sensitive site in the GTF3C3 protein and suggest that the c.1436A>G p.(Tyr479Cys) variant may have differential effects on complex assembly in yeast and human systems.

Consistent with the clinical features of our cohort, neuronal loss of *Gtf3c3* in *Drosophila* induced seizure-like behavior, motor impairment, and deficits in habituation learning. Overall, the phenotypes obtained in the *Drosophila* behavioral assays were consistent with *Gtf3c3* knockdown levels as determined by qPCR. The *Gtf3c3 UAS-RNAi-1* was identified to be the strongest line, followed by *UAS-RNAi-2 and UAS-RNAi-3*. By tuning the activity of the UAS-Gal4 system using more stringent temperatures, increased seizure-like behavior in *Gtf3c3*^RNAi-1^ and *Gtf3c3*^RNAi-2^ animals was demonstrated. Habituation deficits were observed only in *Gtf3c3*^RNAi-3^ because the phenotype was masked by more severe motor phenotypes in *Gtf3c3*^RNAi-1^ and *Gtf3c3*^RNAi-2^ animals. Changing the level of knockdown by manipulating the temperature in the habituation assay was not an option because both higher and lower temperature during developmental stages compromise *Drosophila* jump efficiency. Although a fraction (5/12) patients with mutations in *GTF3C3* present with microcephaly, our ubiquitous *Drosophila* models did not reveal significant differences in brain width or lobe area, suggesting that the observed cognitive and behavioral phenotypes are caused by more subtle cellular morphological changes and/or neuronal dysfunction. This conclusion may also apply to the human condition because severe ID and/or seizures do occur in the 7 nonmicrocephalic patients. Overall, our results are consistent with a role for neuronal *GTF3C3* in cognitive processes, susceptibility to seizures, and motor control.

In summary, we present compelling computational and functional data supporting a causal relationship between rare biallelic missense and splicing variants in *GTF3C3* with an autosomal recessive form of syndromic ID, with variable nonfamilial facial features, severe language delay, motor impairments, seizures, and cerebellar/corpus callosum malformations. Given the strong link between *GTF3C3* and human neurological disorders, other components of the TFIIIC complex represent strong candidate genes for analysis in syndromic ID.

## Supplementary Material

Table S1

Suppl Data

## Figures and Tables

**Table 1:** Summary of variant and clinical features of individuals with *GTF3C3* missense, splicing and indel variants

Family	F1-Germany		F2-Saudi Arabia		F3-Switzerland	F4-Australia	
Individual	P1	P2	P3	P4	P5	P6	P7
GRCh38/hg38 Location	NC_000002.12:g.196776584T>C	NC_000002.12:g.196776584T>C	NC_000002.12:g.196778893T>C	NC_000002.12:g.196778893T>C	NC_000002.12:g.196791369G>A /NC_000002.12:g.196766684G>A	NC_000002.12:g.196791369G>A	NC_000002.12:g.196791369G>A
HGVS cDNA	c.1436A>G	c.1436A>G	c.1390+3A>G	c.1390+3A>G	c.503C>T /c.2419C>T	c.503C>T	c.503C>T
HGVS protein	p.(Tyr479Cys)	p.(Tyr479Cys)	Splice site p. (Pro407_Gln500del)	Splice site (p.Pro407_Gln500del)	p.(Ala168Val) / p.(Arg807Cys)	p.(Ala168Val)	p.(Ala168Val)
TPR domain	TPR8	TPR8	Deletion of TPR7/8	Deletion of TPR7/8	TPR1 / −	TPR1	TPR1
Zygosity	Homozygous	Homozygous	Homozygous	Homozygous	Compound Heterozygous	Homozygous	Homozygous
Age, Sex	13y, F (sib of P2)	22y, F (sib of P1)	F, 5y 9m (sib of P4)	M (sib of P3)	F, 24y	F, 6y (sib of P7)	M, 17y (sib of P6)
Ethnicity	Turkish	Turkish	Saudi Arabian	Saudi Arabian	Swiss/European	Lebanese	Lebanese
Parental	Consanguinity	First cousins	First cousins	Yes	Yes	No	Yes
Yes							
Neurobehavior (ID/DD)	Moderate ID	Mild ID	Severe ID	Severe ID	Severe ID	Severe ID	Severe ID
Nonfamilial facial features	Low frontal hairline, synophrys, overbite, broad nasal bridge, hypertrophic buccal mucosa, single transverse palmar crease right hand, slim fingers, slim feet with hippocratic nails, dry skin with hyperpigmentation on right abdomen, hypertrichosis	Low frontal hairline, thick eyebrows, broad nasal back, wide palpebral fissures, dry skin	Facial asymmetry, bilateral temporal narrowing, epicanthal folds, upslanting palpebral fissures, bulbous nose and full cheeks.	Upslanting palpebral fissure; bulbous nose and full cheeks epicanthal folds	High, narrow palate, abnormality of the hairline, widow's peak, broad eyebrow, macrodontia of permanent maxillary central incisor, diastema, short distal phalanx of finger, overlapping toe, short foot bilateral	Micrognathia, cleft palate, Pierre Robin sequence, broad nasal tip, curved eyebrow	Cleft palate, Pierre Robin sequence, full brow, broad nasal tip, thickened features/full lips
Seizures and age of onset	+ 16 months	+ (onset unknown)	−	+ 1 year	+ 4 years 3 months	−	+ (onset unknown)
Seizure Semiology	Two febrile seizures with EEG abnormalities, a few absences	Absences	N.A.	Tonic	Myoclonic, generalized tonic-clonic, focal impaired awareness, reflex seizures	Absence seizures	Clonic seizures
Current seizure frequency	Seizure free since age 6y 6m	no seizures, but still EEG anomalies at 22y	N.A.	Only once	1-3 focal impaired awareness seizures per month, GTCS rare	Occasional	Nil
Antiepileptic treatment	No medication since age 10y	Ethosuximide	N.A.	Off treatment at the age of 3yrs	Clobazam, Valproate	Nil	Midazolam, Valproate
EEG results	Abnormal in infancy/early childhood	Still abnormal at 22y	Abnormal during wakefulness due to slow background for age. Generalized cortical dysfunction.	Slow background, no clear epileptiform discharges	Diffuse slow and monomorphic rhythm, epileptiform discharges,	Normal EEG at 2yrs	−
Microcephaly	−	−	+	+	+ (secondary)	−	−
Brain Imaging	MRI in Turkey at age 2y reported as abnormal, no details available	−	MRI at 7m prominent cortical sulci, dandy walker variant Aplasia/Hypoplasia of the corpus callosum	Diffuse brain atrophy with thinning of corpus callosum	Corpus callosum hypoplasia at age 8m; progressive, severe cerebral and marked cerebellar atrophy with simplified gyral pattern and hypoplastic frontal lobes bilaterally at age 15y 11m	Cerebellar atrophy, Cerebellar hypoplasia	Progressive cerebellar volume loss, posterior fossa arachnoid cyst and evidence of cortical dysplasia in the insular regions bilaterally
Neurological Features	Ataxic gait, balance problems, sporadically dystonic movement of the hands	N.A.	Head ataxia, hypotonia	Axial hypotonia, spastic quadriplegia	Limb spasticity, spastic dyskinetic tetraparesis, abnormal conjugate eye movement and impaired smooth pursuit, choreoathetotic movement disorder at age 2m to 6m	Dystonia/spasticity, generalized rigidity, pes cavus	Dystonia/spasticity, hyperreflexia, clonus
Gastrointestinal anomalies	+	N.D.	+	+	+	+	N.D.
Other anomalies	Hearing loss	N.D.	−	−	+ Delayed puberty	+	+
Source	Ref 13	Ref 13	Ref 14	This study	Ref 15	This study	This study

Family	F4-Australia	F5-Australia	F6-Netherlands	F7-Egypt/US	
Individual	P8	P9	P10	P11	P12
GRCh38/hg38 Location	NC_000002.12:g.196791369G>A	NC_000002.12:g.196791369G>A	NC_000002.12:g.196779018A>G /NC_000002.12:g.196766683C>T	NC_000002.12:g.196791369G>A	NC_000002.12:g.196791369G>A
HGVS cDNA	c.503C>T	c.503C>T	c.1268T>C / c.2420G>A	c.503C>T	c.503C>T
HGVS protein	p.(Ala168Val)	p.(Ala168Val)	p.(Leu423Pro) /p.(Arg807His)	p.(Ala168Val)	p.(Ala168Val)
TPR domain	TPR1	TPR1	TPR7 / −	TPR1	TPR1
Zygosity	Homozygous	Homozygous	Compound heterozygous	Homozygous	Homozygous
Age, Sex	F, 2y 7m (cousin of P6/P7)	M, 3y	M, 7y	M, 15y (sib of P12)	M, 9y 8m (sib of P11)
Ethnicity	Lebanese	Lebanese	Dutch	Egyptian	Egyptian
Parental	Consanguinity	Yes	Yes	No	First cousins
First cousins					
Neurobehavior (ID/DD)	Moderate ID	Severe ID	Mild ID, clinical suspicion of ADHD	Moderate ID Mild ASD	Moderate ID
Nonfamilial facial features	Almond eyes, curved eyebrows, broad nasal tip, long philtrum, large ears	High forehead, bulbous nasal tip, mild esotropia	High forehead, long eye lashes, blue eyes, down slanting palpebral fissures, small nose, full lips, wide palate, broad jaw	High forehead, bushy thick eyebrows, straight palpebral fissures with almond shape eyes, broad nose and triangular, bulbous tip of nose, flat philtrum, straight lips, broad chin, low set ears	High forehead, bushy thick eyebrows, straight palpebral fissures with almond shape eyes, broad nose and triangular, flat philtrum, straight lips with v shaped upper one, broad chin, low set ears
Seizures and age of onset	+	+ 6 years 2 months	+ 11 months	+ 2 years	+ 3 years
Seizure Semiology	No documented seizures	Staring with tonic posturing of arms	No definitive seizures but suspected seizures in past. Anti-epileptic drugs until 4y	Myoclonic, versive seizures and infrequent generalized tonic	Generalized tonic, myoclonic
Current seizure frequency	N.D.	Several times a week before starting treatment	−	Once per week	Daily (5-8 times)
Antiepileptic treatment	Valproate and midazolam	Valproate	Prior Valproate, Carbamazepine, Depakine	Valproate, Levetiracetam	Valproate
EEG results	N.D.	Fast >30 Hz activity over bilateral frontal and temporal regions, followed by generalized slow rhythmic 2-3 Hz activity in the first seizure and sharp waves with a bifrontal distribution in the second seizure	Normal	Bitemporal epileptogenic discharges at 11 y	Paroxysms of polyspike and slow waves at 9.5 y
Microcephaly	−	+	+	−	−
Brain Imaging	Progressive cerebellar volume loss, posterior fossa arachnoid cyst and evidence of cortical dysplasia in the insular regions bilaterally	MRI pending	MRI cerebrum: megacysterna magna	−	Cerebellar atrophy, thin corpus callosum/12.7y
Neurological Features	Hyperreflexia, dystonia, spasticity and clonus	Limb hypertonia, central hypotonia, foot clonus and lower limb tremors, diagnosis of hypotonic CP	Lower Limb spasticity, myoclonus	Hypotonia, hand tremors, ataxia	Hypotonia, Hand tremors, ataxia
Gastrointestinal anomalies	N.D.	N.D.	+ Constipation	N.D.	N.D.
Other anomalies	−	−	+ Lower Limb spasticity	−	+ Mild ASD, hypotonia
Source	This study	This study	This study	This study	This study

*AR*, autosomal recessive; *ASD*, autism spectrum disorder; *DD*, developmental delay; *EEG*, electroencephalography; *F*, female; *ID*, intellectual disability; *M*, male; m, months; *MRI*, magnetic resonance imaging; *N.A.*, not assessed; *N.D.*, not diagnosed; *P*, patient; *TPR*, tetratricopeptide repeat; *y*, years.

Variants described using the reference sequences *NM_012086.5* (transcript) and *NP_036218.1* (protein).

## Data Availability

This study did not generate data sets or code. All methods are provided in the manuscript or in supplemental files. For data or resource enquiries, please contact Prof Robert J. Harvey, School of Health, University of the Sunshine Coast, Maroochydore, QLD 4556, Australia. rharvey2@usc.edu.au
